# Using a Predictive Risk Model to Prioritize Families for Prevention Services: The Hello Baby Program in Allegheny County, PA

**DOI:** 10.1007/s11121-025-01802-1

**Published:** 2025-04-22

**Authors:** Rhema Vaithianathan, Diana Benavides-Prado, Rebecca Rebbe, Emily Putnam-Hornstein

**Affiliations:** 1 Centre for Social Data Analytics, Auckland University of Technology, Private Bag 92006, Auckland, 1142 New Zealand; 2 School of Social Work, University of North Carolina at Chapel Hill, Chapel Hill, USA; 3 School of Computer Science, The University of Auckland, Auckland, New Zealand

**Keywords:** Predictive risk model, Prevention, Child maltreatment, Home visiting

## Abstract

Population-based efforts to prevent child abuse and neglect are challenging because annual incidence rates are relatively low. Even among families that meet eligibility and risk criteria for intensive home-visiting programs, the baseline rate of maltreatment tends to be low because we use simple criteria. This creates both service (i.e., cost) and evaluation (i.e., power) challenges because a large number of families need to receive the preventive intervention to produce detectable changes in subsequent maltreatment. The increase in the availability of administrative data has made it possible to use predictive risk models (PRMs) to risk-stratify whole birth cohorts and identify children at the highest risk of maltreatment and other early childhood adversities. The current paper describes the development and validation of a PRM implemented in Allegheny County, Pennsylvania, to stratify families and newborn infants into three levels of prioritized services based on the predicted risk of child removal due to maltreatment by age 3. Using a research dataset of anonymized records for children born in Allegheny County between 2012 and 2015, predictive features were coded using data available in the county’s administrative data systems. This spine was linked to child removal outcomes between 2012 and 2018, so we had a 3-year follow-up for each child. A PRM was trained to predict removals in the first 3 years of life using the least absolute shrinkage and selection operator. Predictive accuracy was measured for the highest 5% of risk scores in a holdout dataset. The model was validated using nontraining outcomes such as maternal mortality, infant mortality, and maltreatment-related fatalities and near-fatalities. The model achieved an area under the receiver operating characteristic curve of .93 (95% CI [0.92, 0.95]), recall of 19.93%, and precision of 54.10%. Children identified for the top tier of services had a relative risk ratio of maltreatment-related fatality or near-fatality of 5.54 (95% CI [3.41, 9.00]). Using alternative eligibility approaches (e.g., poverty, teen maternal age) proved far inferior to using PRM in targeting services for children at high baseline risk of maltreatment.

## Background

Child abuse and neglect continues to be a major public health problem. In the USA, approximately 4 million cases of suspected child abuse and neglect involving 7 million children are reported each year to child protection systems (CPS); 600,000 children are confirmed as victims of abuse or neglect, and more than 1,800 fatalities are attributed to abuse or neglect (US Department of Health and Human Services, 2023). Child maltreatment has significant short-term and long-term impacts on physical, developmental, and mental health (Leventhal et al., 2010; Shonkoff, 2016; Widom, 2014). Children who experience maltreatment have lower levels of educational achievement and as adults, they have lower rates of employment, lower earnings, fewer assets, an increased risk of substance abuse, and a higher likelihood to engage in crime and be incarcerated (Cicchetti & Handley, 2019; Currie & Tekin, 2012; Currie & Spatz Widom, 2010; Eckenrode et al., 1993; Lansford et al., 2002; Mersky & Topitzes, 2010; Widom, 1989; Zielinski, 2009).

Federal funding for the prevention of child abuse and neglect, albeit limited, started in the 1990s through community-based child abuse prevention funds (Jones Harden et al., 2020). Most recently, the Family First Prevention Services Act of 2018 sought to increase spending on child maltreatment prevention by expanding the eligibility of Title IV-E funding to include evidence-based clinical interventions for families with a child at risk of imminent out-of-home placement (Testa & Kelly, 2020). Prevention strategies have been categorized at three levels: primary, secondary, and tertiary. Primary prevention services are programs provided to an entire population to prevent initial maltreatment, secondary prevention services are directed to families with CPS involvement but not substantiated for maltreatment, and tertiary prevention responds to maltreatment to prevent recurrence (Scott et al., 2016). In this model, funding is provided for tertiary prevention services that mitigate the harms that triggered CPS involvement, preventing the placement of the child as opposed to preventing maltreatment (Testa & Kelly, 2020). Beyond these two sources, federal funding for child maltreatment is primarily reactive as opposed to preventive, and child maltreatment prevention services typically occur outside of CPS (Berger & Slack, 2020).

Primary prevention of child maltreatment is challenging, and rigorous evaluation of programs is limited (Eckenrode, 2011; Jones Harden et al., 2020). For one thing, low enrollment and completion rates hinder the population-level efficacy of maltreatment prevention programs and other voluntary parenting interventions (Gonzalez et al., 2018; Winslow et al., 2018), especially among high-risk or high-need families (Alonso-Marsden et al., 2013; Dumas et al., 2007). A systematic review (Levey et al., 2017), limited to randomized controlled trials of interventions to prevent child abuse and neglect, found that only two evaluations provided statistically significant reductions in child maltreatment: Nurse-Family Partnership (NFP) and Healthy Families New York (HFNY). Both NFP (DuMont et al., 2008) and HFNY (Olds et al., 1986) focus on low-income and first-time mothers. The programs provide prenatal visits, biweekly postpartum visits, and less frequent visitation through the child’s second birthday. They differ in that professional public health nurses provide NFP services, whereas HFNY relies on lay visitors. Despite early successes, contemporary implementations of NFP have failed to replicate the same long-term preventive effects (McConnell et al., 2022) and subsequent Healthy Families America programs have produced inconsistent results (Duggan et al., 2004; Green et al., 2017). One challenge to implementing maltreatment prevention programs outside of CPS is the paucity of studies on very high-need families whose children may later experience maltreatment. Families at risk of maltreatment often struggle with serious substance abuse, mental health, and interpersonal violence (Palmer et al., 2024). A recent evidence review of programs for mothers facing adversities noted:

Many parents face two or more of these challenges, and some face nearly all of them. There has been almost *no rigorous evaluation* [emphasis in original] of interventions for these very complex cases, and many of these families are referred to child welfare agencies. (National Academies of Sciences, Engineering, and Medicine, 2016, p. 251)

A second challenge to the primary prevention of maltreatment is the ability to identify and engage families whose children have sufficiently heightened risk. The increase in the quality and availability of administrative data in recent decades has made it possible to implement new tools for classifying risk, including predictive risk models (PRMs). PRMs can automatically generate a risk score for all individuals with information in a data system, providing a cost-effective means of population risk screening without requiring additional data entry by clinicians or other staff members. PRMs have been widely discussed and used in healthcare (Obermeyer & Emanuel, 2016), with more recent applications in human services (e.g., homelessness services; Kithulgoda et al., 2022) and CPS (Drake et al., 2020; Rosholm et al., 2024). In a more recent illustration of how PRMs could improve targeted prevention efforts, Ahn et al. (2024) showed that an algorithm applied to birth records successfully identified more than 75% of children who later experienced maltreatment. The PRM was significantly better than the baseline model being used to identify families that might benefit from home-visiting services.

During the past few decades, the Allegheny County Department of Human Services (DHS) has developed an integrated data warehouse across service systems. In 2016, the DHS began developing, in partnership with local providers, a prevention program called Hello Baby. This program universally screens the county’s population of newborns to identify those with a heightened baseline risk of maltreatment; a pilot implementation of the program launched in September 2020. Hello Baby is unique in that it uses a PRM to stratify families and their newborn infants into three levels of services based on information in the county’s integrated data warehouse. All families are eligible for some level of support, but higher-priority families (i.e., where primary prevention programs may have the highest impact) are eligible for more intensive services and proactive outreach and engagement by community providers. The DHS worked with a research team to develop the Hello Baby PRM, an algorithm run shortly after each birth in the county. The model is designed to identify newborns with the greatest risk of removal from the home due to maltreatment by their third birthday.

This paper reports on the development and validation of the Hello Baby PRM for Allegheny County. We describe the data used to code features used in the model training process; modeling decisions made to develop the PRM subsequently implemented by the county; predictive accuracy of the model when applied to holdout records not used in the training process; and efforts to validate the PRM externally across adverse outcomes measured in administrative data but not used to train the model. The paper concludes with a discussion of the implications for primary maltreatment prevention programs and the challenges and limitations of this type of real-world prioritization of families for services using data-driven approaches.

Our paper can be situated in the literature on PRMs in child welfare, first developed using birth data from a New Zealand cohort (Vaithianathan et al., 2013). The methodology used in that paper is similar in that it also used administrative records linked to birth records, with a predicted outcome of substantiation (rather than removal). However, that study used logit regression rather than least absolute shrinkage and selection operator (LASSO) as the statistical approach. The subsequent paper by the same primary author (Vaithianathan et al., 2018) also using New Zealand data showed that a model trained to predict child welfare outcomes also could predict maternal and child adversities, including fatalities. Unlike the Hello Baby model, the New Zealand model was never implemented.

In contrast, the Hello Baby model is fully operational in Allegheny County. Mothers are informed at birth that their administrative data will be used to determine their eligibility for additional programs. At the birthing hospital, where they receive information about the program, they also receive a postcard that they can send to the DHS if they wish to opt out. The Hello Baby PRM is run 6 weeks after the baby’s birth. More details on the model’s operationalization are available in Vaithianathan et al. (2020).

## Method

To develop the PRM, a research dataset was built using anonymized records for children born in Allegheny County between 2012 and 2015. Birth records were used to define a cohort of children born during this 4-year period whose mothers resided in the county (*N* = 52,520). Predictive features were coded using integrated data available in Allegheny County’s data warehouse. Features included characteristics of the child and mother as identified in birth records, along with data on fathers if paternity is established. In addition, features from other universal data systems in the county’s data warehouse (e.g., CPS, homelessness services, criminal justice) were considered, including maternal, paternal, and sibling data. No physical or behavioral health data were incorporated because they are available only for individuals who receive government-funded, rather than private, services. Encrypted client keys at the person and family level were available in the research dataset extracted from the county’s data warehouse.

The Hello Baby PRM was trained to predict CPS removal due to maltreatment before a child’s third birthday. Removal was treated as a proxy for actual, albeit typically unobserved, child abuse and neglect. Removal due to maltreatment was selected as the training outcome for the PRM for two reasons: (a) it reflects an outcome the Hello Baby intervention was designed to reduce (i.e., early childhood maltreatment); and (b) it was not purely a community-generated outcome such as allegations of abuse and neglect nor a system-generated determination such as substantiation, but instead an outcome only observed with court and legal system involvement.

Research data were partitioned into a 70–30 split: a training set containing 39,365 records and a testing set of 13,155 holdout records. Siblings and half-siblings were blocked such that they were always assigned to either the training or test set. This ensured that families in the test set were not also observed in the training data. Individuals who died during the outcome period were excluded from the training data, given they were not at risk of removal.

The PRM was estimated using the R package glmnet (Friedman et al., 2021). The lambda parameter was tuned through a tenfold cross-validation procedure repeated three times, yielding three random partitions of the original training set—a standard practice in machine learning (Hastie et al., 2009; Witten & Frank, 2005). Those results were again averaged to produce a single estimation. Among 100 lambda values tested, the model that corresponded to the best lambda was considered the final model.

The model was trained using the LASSO regularized logistic regression algorithm. LASSO ensures certain predictor weights are set to zero while minimizing prediction error, given the sum of the absolute value of the weights is less than a constant. Thus, it is capable of both predictor selection and regularization, resulting in more easily interpretable and accurate models.

In the current context, two primary classification mistakes can be made by a PRM. The first is to identify a newborn and their family as having a high need for services, even though this family would not have had adverse outcomes regardless of services. This type of error (i.e., false positive) may lead to the overprovision of services. Although unlikely to be harmful to these families, these types of errors increase inefficiency when intensive service slots are limited, potentially meaning children with higher needs go unserved. The other type of classification mistake occurs when the model fails to identify a newborn and their family as having high need (i.e., false negative). In these situations, families mistakenly receive a lower tier of services than they need, and because those services are inadequate, the child might experience preventable adversities. The failure to classify a family with high needs properly means that the county misses an opportunity to prevent harm.

The LASSO was tuned to maximize the area under the receiver operating curve characteristic (AUC). The benefit of tuning to AUC is that it evaluates the PRM’s ability to rank individuals. Because the program offered has graduated intensity, it relies on the PRM to rank families across classification thresholds.

The Hello Baby PRM was developed to provide a data-driven means for the county to direct prioritized access to the most intensive services to families with the most complex needs. To test if children identified by the PRM tool as needing the highest tier of services truly had high risk of adverse outcomes, broad validation outcomes not used as the training outcome were extracted from the county’s data warehouse. These measures were coded as occurring if the child (or their mother) experienced the event within 3 years of birth. Given that missing paternity varied by risk level, we did not attempt to conduct validations based on paternal outcomes. Although we were restricted to measuring adversities observed in administrative data, the rich array of system data available in the Allegheny County data warehouse meant that we could observe and validate our model in the context of many adverse outcomes (e.g., homelessness, arrest). Included in these validation outcomes were three child mortality rates: (a) all-cause mortality, (b) postneonatal infant mortality, and (c) mortality with a code of injury or near-fatality due to maltreatment investigated under Pennsylvania state requirements. Near-fatal and fatal events are rare—which necessitates caution about interpreting exact numbers—but they are the best “ground truth” measures of harm in administrative data systems. We present the prevalence of each outcome, along with a more detailed definition, in Table 1.

**Table 1:** Outcomes used to train and validate model

Outcome observed after birth of child	Definition	Prevalence in research data (per 1000)
Child removal within three years of birth (training outcome)	Child had at least one removal due to risk or safety concerns by CPS	18.13
Case opening by CPS	Mother had a case opened by CPS for services	37.47
Maternal homelessness	Mother met criteria for receipt of homelessness services	7.52
Maternal jail booking	Mother had at least 1 night in Allegheny County Jail	9.73
Maternal mortality	Mother identified as a decedent in death records held by Allegheny County	0.42
Child mortality	Child identified as a decedent in death records held by Allegheny County	6.15
Postneonatal infant mortality	Child identified as a decedent with an age at death between 1 and 12 months	1.60
Violent, accidental, or maltreatment-related child mortality or near-mortality	Child recorded in death records or as a near-fatality due to violence, accident, or maltreatment that required review under Pennsylvania law	1.77
Maternal behavioral health^a^	Mother recorded as having received substance use or mental health services	56.86
Maternal emergency room encounter^a^	Mother recorded as having had an emergency room visit	226.27

Mortality established using vital death records derived from Allegheny County’s Department of Public Health. Due to lags in certifying official death records, we cannot establish that all child or maternal death events have been included and therefore, mortality rates reported here may be lower than the true population prevalence. The sample size was 52,520

^a^Outcome observed only for mothers enrolled in Medicaid. The sample size for Medicaid-eligible mothers was 14,562

For each validation measure, we undertook a relative risk calculation, comparing the rates of those prioritized for Hello Baby services (i.e., top 5% of risk) with those of children in the other 95% of the sample. Although we used R to build the model, for the estimation of relative risk and confidence intervals, we used Stata version 16.0.

## Results

Table 1 provides summary details about the research data, which represented children born between 2012 and 2015. The research dataset used to develop the PRM featured 52,520 births. The average maternal age at birth was 29.3 years; 6.5% of mothers had less than a high school education; and 21.0% identified as Black, 72.9% identified as White, and 1.9% identified as Hispanic. A sizeable proportion of births did not have paternity established and, thus, did not have associated paternal information (13.9%–15.6% missing). For those with paternal information, the average age of fathers was 32.0 years; 0.4% had less than a high school education; 15.8% identified as Black, 64.3% identified as White, and 2.0% identified as Hispanic. Overall, 44.8% of births were first births and 32.9% were second births.

Table 2 describes the prevalence of all outcomes examined, including both child removal (used for PRM training) and other outcomes used for model validation. The overall removal rate by age 3 was 18.13 per 1000 births. Case openings for the child (or sibling) by CPS occurred at a rate of 37.47 per 1000 births. Child mortality by age 3 was 6.15 per 1000 births, whereas near-fatalities and fatalities were observed for 1.77 per 1000 births. Maternal outcomes are presented for the birth population overall (e.g., maternal homelessness was 7.52 per 1000) and mothers enrolled in Medicaid (e.g., maternal emergency room visits were observed for 226.27 per 1000 Medicaid births).

**Table 2:** Characteristics of the sample

Category	Mother	Father
Age at birth (*M*)	29.3	32.0
Age missing (%)	< 0.5	13.9
Education less than high school (%)	6.5	4.4
Education missing (%)	< 0.5	14.5
Black race (%)	20.9	15.8
Hispanic ethnicity (%)	1.9	2.0
White race (%)	72.86	64.27
Race and ethnicity missing (%)	1.63	15.63
Child birth year		
2012	12,915	
2013	13,156	
2014	13,211	
2015	13,238	
First birth (%)	44.8	
Second birth (%)	32.9	
Total births	52,520	

Table 3 shows the predictive accuracy of the Hello Baby PRM for the training outcome of removal by age 3. The analysis was restricted to the random subset of 13,155 births not used in the training of the PRM (i.e., test set). The Hello Baby program defines high risk as prediction scores in the top 5% of all births and designates these families for priority services. Therefore, we report an accuracy measure regarding this top 5% group. AUC, recall, and precision are all indicators of classification accuracy. The rate of removals in the first 3 years for the Hello Baby priority group was estimated to be 19.9%—and this group accounted for 54.0% of all children in the test sample removed before age 3.

**Table 3:** Recall, precision, and AUC of Hello Baby PRM predicting removals (test set only)

Measure	Statistic
AUC (95% CI)	0.93 (0.92, 0.95)
Removal rate among top 5% of risk group (recall)	19.9%
Share of all removals identified in top 5% (precision)	54.1%
Sample size of test data	13,155

**Fig. 1:**
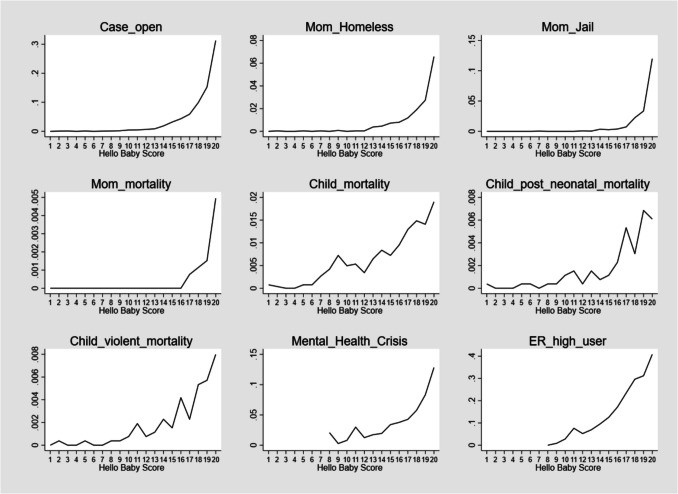
Prevalence of validation outcomes against the Hello Baby Predictive Risk Model Score (measured in 20 ventile score bins)

Figure 1 plots the prevalence of the training and validation outcomes (defined in Table 2) against Hello Baby PRM scores. The PRM scores represent 5% divisions of the scored population. For instance, children in the lowest 5% of predicted risk received a score of 1, whereas children with the highest 5% of predicted risk received a score of 20. The plots consistently document a strong, graded relationship between the scores and the training and validation outcomes. Although some outcomes, such as having a case opened for services, may be mechanically associated with the predicted outcome of removal (i.e., case openings are a common precursor to removal), other outcomes such as near-fatality or fatality are best understood as “ground truth” adversities that primary prevention programs should effectively address.

Table 4 shows the relative risk associated with being classified in the top 5% for each validation outcome. Children identified for Hello Baby priority services were 5.5 times (95% CI [3.41, 9.00]) as likely to be involved in a maltreatment near-fatality or fatality. Given the focus of Hello Baby on the primary prevention of maltreatment, this confirms that even though the PRM was trained to predict removals, it also identifies young children at considerably heightened risk of severe harm. The relative risk of other outcomes was also high, ranging from 2.1 for maternal emergency room encounters to 30.6 for incarceration.

**Table 4:** Relative risk of validation (nontraining) outcomes

Outcome	RR (95% CI)^a^
Case opening	13.63 (12.57, 14.77)
Homelessness	14.81 (12.18, 17.99)
Maternal jail	30.54 (25.66, 36.34)
Maternal mortality	27.44 (11.74, 64.15)
Child mortality	3.48 (2.58, 4.69)
Postneonatal infant mortality	4.47 (2.60, 7.70)
Violent, accidental, and maltreatment-related child mortality or near-mortality	5.54 (3.41, 9.00)
Maternal mental health crisis^a^	2.96 (2.59, 3.39)
Four or more maternal emergency room visits^a^	2.13 (2.00, 2.26)

Column 2 shows the relative risk of having the outcome among those predicted to be in the top 5% of risk compared with the rest (95%) of the cohort. The sample size was 52,520, with 2626 in the top 5%

^a^Outcome observed only for mothers eligible for Medicaid at the child’s birth (total sample of 14,562, in which 2322 scored in the top 5%)

**Fig. 2:**
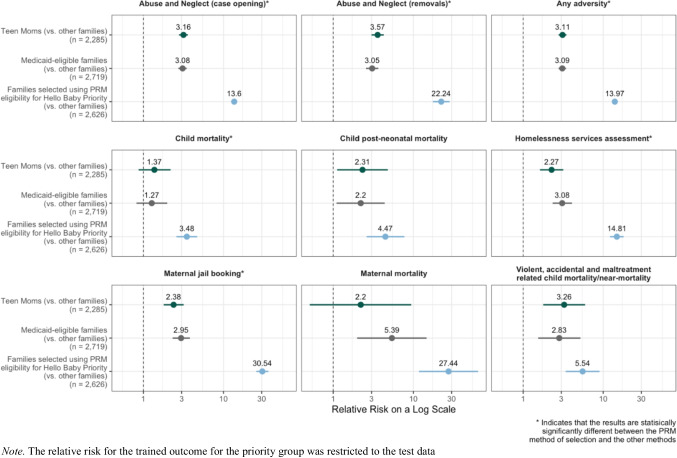
Relative risk of adverse outcomes for counterfactual approaches to determine eligibility

Finally, a potential objection to using a complex algorithmic strategy to identify families for intensive support is whether prioritization could be established based on simpler rules, such as poverty (as proxied by Medicaid status) or young maternal age, with equal effectiveness. Figure 2 presents recalculated relative risks for all adverse outcomes using two alternative methods for identifying 5% of a birth cohort for Hello Baby priority services. These rules included 2719 randomly selected Medicaid-eligible families (Column 2) or maternal age less than or equal to 19 years (Column 3). Column 1 of Fig. 2 reproduces the information in Table 4 and provides the relative risk based on the PRM. Across the board, the simplified alternative methods for selecting roughly 5% of the birth cohort were far less refined in identifying children and families most likely to experience adversities. For example, the relative risk of maltreatment-related fatality ranged between 2.8 and 3.3, compared with 5.5 using the PRM.

## Discussion

The current study tested and validated a PRM used to screen and identify newborns and families for Allegheny County’s most intensive support and services. At least three important findings emerged with implications for child maltreatment prevention, alongside broader insights relevant to the efficient deployment of population-based prevention programs. First, the findings presented here indicate that it is possible to use administrative records to passively screen populations of newborns and identify families with a notably heightened risk of removal due to child abuse and neglect. The use of a PRM requires no new data collection and has incredibly low costs relative to all other screening approaches. The Hello Baby PRM shows that a predictive model trained to estimate the probability of child removal by age 3 can be used to identify children and families with 20 times the risk of early childhood removal due to child maltreatment relative to other newborns. The findings are in line with previous work with a New Zealand birth cohort in terms of accuracy in predicting both child welfare involvement (substantiation in that case) and a broader range of outcomes using administrative data (Vaithianathan et al., 2013, 2018).

Second, the Hello Baby PRM demonstrated a strong, graded relationship to not only the outcome it was trained to predict (i.e., child removal due to maltreatment) but also other adversities. Importantly, these adversities included more direct measures of objective child harm, such as maltreatment near-fatalities and fatalities. The relationship that emerged is notable because it underscores that although local base rates of physical harm to children may be low and therefore, a poor target for model training, they can serve as important tools in the subsequent validation of a PRM trained to predict a system outcome. Meanwhile, the graded relationship between PRM scores and maternal adversities highlights the complex nature of maltreatment prevention.

Third, comparisons to simple, rule-based methods indicated that a PRM is a far more accurate (and refined) means of prioritizing families for prevention services. Across all adverse outcomes examined, the PRM method for choosing 5% of births to prioritize for services was dominant. Given that most jurisdictions are constrained in the number of families that can be recruited for and served by a resource-intensive prevention program, choosing families based on simple indicators of poverty or demographic characteristics is likely inefficient relative to a PRM.

## Limitations and Challenges

Although this study explored an implemented PRM, one challenge to scaling tools such as the Hello Baby PRM is the availability of data for training, validation, and deployment. Indeed, Allegheny County is unique among human services agencies in the USA in its longstanding investments in an integrated data warehouse and adoption of data-driven practices. That said, as public agencies improve their data systems and technologies, it seems likely that PRMs to support more targeted prevention efforts will follow. Beyond technical considerations, another barrier to the effective use of PRMs is gaining the social license required for government agencies to use administrative data for proactive recruitment of families into services; thoughtful program design is critical. In Allegheny County, all families are informed at birth that their data may be used to identify their eligibility for additional support through Hello Baby. Families then have time to opt out of an eligibility screening. After this window has passed and the county receives the birth certificate, the PRM screening assessment is performed. Community-based providers are responsible for contacting and engaging with families. At that point, families must consent to enroll and interact with service providers, similar to standard prevention programs.

A major limitation is the lack of generalizability of our findings to other regions. Because we have used administrative data from a specific location, the same level of predictive accuracy might not be possible using similar data in another setting. For example, jurisdictions with substantial movement across borders might have significant attrition as families move in and out of their region.

We are also limited by the proportion of births that did not have paternity established and thus, lacked associated paternal information (13.9–15.6% missing). This means that we could not validate predictive accuracy when including all paternal predictors, and we expect that more complete information from paternal history would have greatly improved the predictive accuracy.

## Conclusion

Prevention programs must choose the right candidates to be effective. Traditionally, primary prevention programs for maltreatment have selected candidates using simple, rules-based criteria. As shown here, such targeting regimes often fail to identify families with the highest risk of maltreatment. Using administrative data, a PRM can identify candidates at much higher risk of various outcomes.

If the availability of intensive prevention slots is constrained, then the potential cost-effectiveness of a PRM is 7 times that of using an indicator of poverty. Of course, this assumes that prevention programs are effective for these extremely high-risk families. Having found these high-need families, Allegheny County officials are currently designing a program that serves their needs. Because families recruited by the PRM have far greater needs, often across multiple domains, services must be highly responsive and coordinated across health care, housing, and child welfare services. The evolving nature of the program in Allegheny County also makes evaluation a challenge—and clearly the next step in the journey.

## Data Availability

The data is available upon request to Allegheny County, PA. See https://www.alleghenycountyanalytics.us/requesting-data/.

## Appendix

Table 5

**Table 5:** Data sources used for training and validating the model

Validation outcome	Definition	Source data
Abuse and neglect (case opening)	Case opened for child welfare service by age 3	DHS children, youth, and family services records
Homelessness Services assessment	Any interaction by Mother recorded with the LINK call center that assesses people for homeless services; and where they met eligibility within 3 years of birth	DHS homelessness and housing services records
Maternal jail booking	Had a booking in Allegheny County Jail within 3 years of birth	Allegheny County Jail data
Maternal mortality	Maternal death observed to the end of the period for which death records were linked	Vital death records
Any of the adversities listed above	At least one of the adversities listed above (maltreatment, homelessness, maternal jail booking, maternal mortality) used for validation purposes	As above
Child mortality (any cause)	Any cause child’s death up to end of the period for which death records were linked	Vital death records
Violent, accidental, and maltreatment-related child mortality or near-mortality	A combination of child death with a coding of injury, drowning, or other preventable reason and referral to the state for fatality or near-fatality caused by maltreatment within 3 years	Vital death records and county referrals under Act 33
Child postneonatal mortality	Child’s death due to any cause during the postneonatal period (28 days to 1 year)	Vital death records
Maternal behavioral health crisis (Medicaid-funded only)	Medicaid funded mental health crisis services used within 3 years of birth	Medicaid data
Maternal emergency room visits for medical care (Medicaid-funded only)	Four or more Medicaid funded emergency room visits for maternal medical care within 3 years of birth	Medicaid data
